# Understanding Supporting and Hindering Factors in Community-Based Psychotherapy for Refugees: A Realist-Informed Systematic Review

**DOI:** 10.3390/ijerph17134618

**Published:** 2020-06-27

**Authors:** Douglas Gruner, Olivia Magwood, Lissa Bair, Liezl Duff, Shiva Adel, Kevin Pottie

**Affiliations:** 1 Department of Family Medicine, University of Ottawa, 600 Peter Morand Crescent Suite 201, Ottawa, ON K1G 5Z3, Canada; shiva.adel@gmail.com (S.A.); kpottie@uottawa.ca (K.P.); 2 C.T. Lamont Primary Health Care Research Centre, Bruyère Research Institute, 85 Primrose Ave, Ottawa, ON K1R 6M1, Canada; omagwood@bruyere.org (O.M.); liezlvinci.ca@gmail.com (L.D.); 3 Faculty of Medicine, University of Ottawa, Roger Guindon Hall, 451 Smyth Rd #2044, Ottawa, ON K1H 8M5, Canada; lbair034@uottawa.ca; 4 School of Epidemiology and Public Health, University of Ottawa, 600 Peter Morand Crescent Suite 101, Ottawa, ON K1G 5Z3, Canada

**Keywords:** global mental health, refugees and asylum seekers, primary health care, NET, CETA, CBT

## Abstract

Culture, tradition, structural violence, and mental health-related stigma play a major role in global mental health for refugees. Our aim was to understand what factors determine the success or failure of community-based psychotherapy for trauma-affected refugees and discuss implications for primary health care programs. Using a systematic realist-informed approach, we searched five databases from 2000 to 2018. Two reviewers independently selected RCTs for inclusion, and we contacted authors to obtain therapy training manuals. Fifteen articles and 11 training manuals met our inclusion criteria. Factors that improved symptoms of depression, anxiety, and PTSD included providing culturally adapted care in a migrant-sensitive setting, giving a role to other clinical staff (task-shifting), and intervention intensity. Precarious asylum status, constraining program monitoring requirements, and diverse socio-cultural and gender needs within a setting may reduce the effectiveness of the program. Primary care programs may enable community based mental health care and may reduce mental health-related stigma for refugees and other migrants. More research is needed on the cultural constructs of distress, programs delivered in primary care, and the role of cultural and language interpretation services in mental health care.

## 1. Introduction

There are 25.9 million refugees in the world [1]. As refugees integrate, more community clinicians are providing global mental health care. Exposure to violence and forced migration increases the risk for common mental health disorders, chronic pain, and other somatic complaints [2]. Global mental health research considers disease prevalence, traditional beliefs, idioms of distress and stigma [3]. Pharmacotherapy may also play an important role in common mental health disorders [2]; however, this study focuses on community-based psychotherapy and trauma-informed care programs (see Table 1).

Refugees do not easily engage in psychotherapeutic relationships [4,5]. Front line physicians are however frequently consulted [6,7]. Refugees prefer community-based care, as opposed to specialist/in hospital care, but are less likely to accept mental health services compared to non-migrant populations [8,9]. Culture impacts patterns of coping, help seeking, adherence to treatment, emotional expression as well as relationships with clinicians [10]. Culture and language interpreters are integral to help clinicians understand the patient’s context [11].

Primary health care clinics can be referred to as the “patient’s medical home”. This “medical home” is a community based interdisciplinary clinic in which patients can access primary healthcare services on a regular basis [12]. While refugee patients visit their “medical home” for medical needs, they often consult for psychosocial needs. For example, refugees are often faced with social issues such as housing or food insecurity [13], and will visit their “medical home” for assistance. These visits for social assistance also allow the primary care clinician further insight into the struggles their patients may be facing, which allows them the opportunity to screen for mental health concerns [2]. These “medical homes” become a trusted space for refugee patients and integration into these “homes”, we argue, is an important element of health settlement and global mental health care. Given this, primary care clinicians may be well positioned to screen, diagnose, and treat common mental health conditions in refugee patients [2].

Interest in community-based psychotherapies for refugees continues to grow [14]. Community based mental health interventions, however, are culturally complex, multi-faceted and dynamic in nature. Consequently, we conducted a realist-informed systematic review to address the following research question: What factors determine the success or failure of community-based psychotherapy for trauma-affected refugees?

## 2. Materials and Methods 

We conducted a realist-informed review to better understand community-based psychotherapies for refugee mental health programs. A traditional realist synthesis is a resource intensive [15], theory-driven approach that seeks to unpack the mechanism of how programs work in particular contexts and settings [15,16]. Several groups have developed pragmatic “realist-informed” reviews. We adopted the approach by Greenhalgh et al. [17], which used a realist lens to analyze rigorous randomized controlled trials. We report our findings according to the RAMESES (Realist and Meta-narrative Evidence Syntheses: Evolving Standards) publication standards (see Supplementary S1) [18].

### 2.1. Research Team and Initial Scoping of the Literature

We assembled a research team consisting of three primary care clinicians specializing in refugee health, one medical student, one international medical graduate, and one research methodologist experienced in reviews of refugee health. We scoped published medical literature for reviews and trials of psychotherapies. Within the team, we discussed these interventions’ reach, effectiveness, adoption, implementation, and maintenance [19]. Through discussion, we applied a realist lens and developed an evaluation matrix that considered participant, provider and intervention characteristics, process details, historical context, and effectiveness outcomes. This matrix informed our subsequent search strategy, data extraction, and analysis.

### 2.2. Search Strategy and Selection Criteria

We included any randomized controlled trial whose population included refugees or asylum seekers with experience of trauma in any geographic context. By trauma, we mean the result of “an event, series of events, or set of circumstances that is experienced by an individual as physically or emotionally harmful or threatening and that has lasting adverse effects on the individual’s functioning and physical, social, emotional, or spiritual well-being” [20]. In the case of refugees, this is often the result of war and/or persecution. We focused exclusively on randomized controlled trials because they are designed around a clear hypothesis; their design minimizes the risk of confounding, and their effectiveness findings are likely to be closer to the true effect than the findings generated by other research methods [21]. We included male or female populations over the age of 18 years who participated in a psychotherapy delivered in any clinical or community setting. We excluded studies focused on children or adolescents and group therapies. We considered any type of comparator (such as waitlist-control or an alternative intervention) and included mental health outcomes of interest: PTSD, depression, or anxiety symptomatology (see Supplementary S2 for full inclusion and exclusion criteria).

We developed a systematic search using relevant keywords and MeSH terms for relevant published randomized controlled trials. Keywords included “refugee”, “asylum seeker”, “trauma”, “PTSD” and “psychotherapy” (see Supplementary S3 for search strategy). We searched MEDLINE, EMBASE, PsycINFO, CENTRAL and PILOTS from 1 January, 2000, to 26 September, 2018. There were no language restrictions. We manually searched reference lists of identified systematic reviews for relevant citations and cross-referenced it against our original search results. Any additional potentially relevant citations were screened. We contacted the authors of all included studies to obtain intervention training manuals or protocols. These training manuals and protocols were included in our review to better understand the theoretical underpinnings and implementation considerations of each identified psychotherapy. 

We uploaded search results to Rayyan reference manager software to facilitate the study selection process [22]. Two review authors independently assessed each study for inclusion by title, abstract and full text. Disagreements were resolved through discussion with a third reviewer. 

### 2.3. Data Analysis

We developed a standardized data extraction template which included study design, setting, participant characteristics, intervention, provider, language, process details, study history, outcomes, mechanisms, and conclusions. We extracted data in duplicate and resolved disagreements through discussion. We assessed the methodological rigor of included studies using the Cochrane Risk of Bias tool in duplicate [23]. The review team discussed the relevancy of each included article to the purpose of the review. An appraisal deemed “low” was used for articles that did not include any information or discussion of mechanism or contextual factors. A rating of “medium” was given to studies that provided information on either contextual or mechanism variables. A rating of “high” was given to studies that provided information on both mechanism and contextual variables. We did not exclude any articles based on our critical appraisal (see Supplementary S4). We followed a realist analysis approach (see Table 2). Chains of inference and hypothesis formulation were developed iteratively through discussion with the entire review team. We discussed preliminary conclusions and synthesized key findings using a narrative and interpretive approach.

## 3. Results

**Figure 1:**
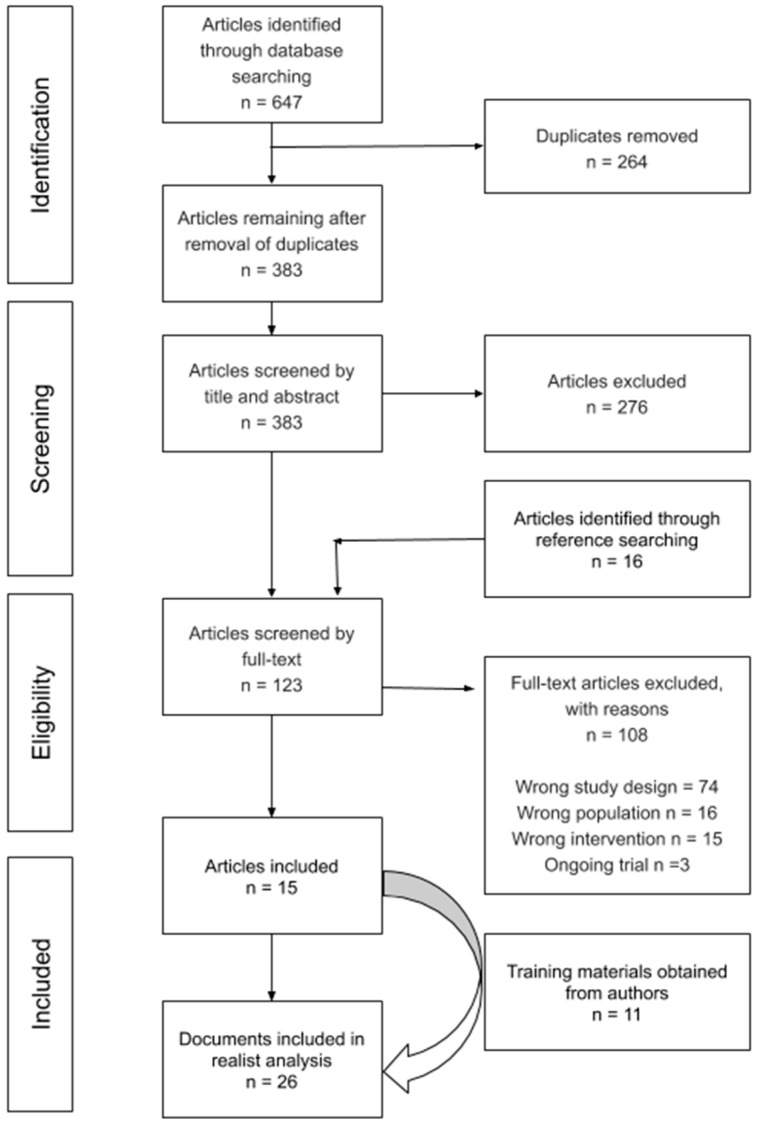
PRISMA flow diagram.

Our systematic search identified 647 citations. After the removal of duplicates, two reviewers independently assessed 383 articles by title, abstract, and full-text review. Of the 123 articles assessed at full-text, 15 articles met the eligibility criteria. We contacted the authors of the 15 articles and included 11 additional training documents in our realist analysis (see Figure 1: PRISMA).

Our 15 included articles captured findings from 14 randomized controlled trials conducted in Denmark (1), Egypt (1), Germany (3), Norway (1), Sweden (1), Thailand (1), Uganda (2), and the United States (4). All studies were conducted among refugees or asylum seekers with experience of trauma. Interventions included Narrative Exposure Therapy (NET), Common Elements Treatment Approach (CETA), Stress Management (SM), Cognitive Behavioral Therapy (CBT), and Interpersonal Therapy (IPT). We obtained training materials for all interventions in the form of books, training manuals, and peer-reviewed articles. See Table 3 for additional study characteristics. 

We tabulated the effectiveness of individual psychotherapies on participant attrition, anxiety, depression, and PTSD symptomology in Supplementary S5. In summary, all included psychotherapies (CBT, CETA, IPT, NET, and SM) had statistically significant positive effects on symptoms of PTSD. Findings on depression and anxiety outcomes varied across studies. For example, four studies on CBT consistently demonstrated improved symptoms of anxiety and depression when compared with a wait-list control [37,40,41] or exposure therapy [49]. In contrast, NET reduced symptoms of depression in three studies [26,36,50] but had no impact on depression in two other studies [35,46]. Researchers highlighted that refugees with a secure legal status reported less depression across all time points [50]. NET was reported to be equivalent to supportive counselling and psychoeducation according to indicators of depression and anxiety [48]. CETA was effective in reducing symptoms of depression and anxiety, and these results were not dependent on the gender of the participant or severity of trauma [29].

Our systematic search and realist-informed analysis of randomized controlled trials provided data which we then applied to a context-mechanism-outcome configuration to evaluate the success of community-based psychotherapy for trauma-affected refugees. The context within which most refugees seek care is generally in migrant-sensitive healthcare settings and community-based practices [2]. These practices improve access, lessen transportation and financial and migration status needs, and reduce mental health treatment related stigma with interdisciplinary primary care teams. Literature suggests that the way in which care is delivered, or the mechanisms of delivery, are fundamental to successful outcomes. In this study we found that the management of refugee mental health can be tasked-shifted from specialty care (e.g., psychiatry) to primary care [29,42,47,48]. Primary care may include physicians, nurse practitioners, nurses, social workers, settlement workers, and sometimes cultural navigators and even shared mental health care teams. Mental health programs and clinicians can adopt a trauma-informed approach and deliver culturally appropriate psychotherapy to refugees with common mental health conditions. Existing psychotherapeutic approaches, such as CBT, have been culturally adapted with success [40,41].

Programs and clinicians need to be aware of socio-cultural preferences and global mental health presentations in refugee patients. Provision of programs, mentorship, advocacy, and psychotherapy of sufficient intensity can empower and enhance self-efficacy, emotional regulation, and social support, thereby improving mental health outcomes.

The theories underpinning the success or failure of community-based psychotherapy for trauma-affected refugees is explored below.

### 3.1. Theories That Explain Why Community-Based Mental Health Services for Refugees Work

#### 3.1.1. Practice: Migrant-Sensitive Healthcare Settings

Whether caused by social, structural or financial barriers, evidence suggests that refugees do not access health services effectively, ultimately impacting health outcomes [29,35,42,46,47]. Refugee patients possess complex medical issues and require special considerations to meet their mental health needs, beginning with the setting of care. Ten studies from high resource settings administered mental health interventions in an outpatient setting [26,29,31,35,37,40,41,46,50,51]. In low resource settings, researchers made use of participants’ homes or quiet places nearby [47], straw huts or under trees [48], and church or community center [36]. In the majority of studies, the setting of care was specialized for general refugee populations [26,35] or a specific ethnic or cultural group such as a Burmese-run clinic for Burmese refugees in Thailand [29] or a Sudanese-founded NGO in Egypt [42]. In such cases, the clinical practice setting addressed social determinants of health and offered migrant-sensitive services including language interpretation, culturally tailored support programs, and community-based cultural support staff.

#### 3.1.2. Provider: Task-Shifting

Task shifting holds promise for improving mental health care delivery in primary care settings. It entails the shifting of tasks, typically from more to less highly trained individuals, to make efficient use of resources. Several included studies showed that some therapies could be adopted by other traditionally non-medical personnel including social workers, lay counselors, teachers, and even former refugees with the appropriate training [29,42,47,48]. However, the majority of psychotherapies were delivered by psychiatrists, clinical psychologists and experienced mental health workers in high income countries [26,31,35,36,37,40,41,46,49,50]. Qualifications for laypersons recruitment included language fluency (English and mother tongue); educational attainment (attended primary school and/or secondary school); interest in mental health/counseling; and shared cultural, religious, and political experience [29,42,47,48]. Of note, none of the studies looked at primary care clinicians delivering these psychotherapies. However, successful task-shifting to non-medical personnel suggests that primary health care clinicians could equally deliver the therapy, and this would likely lead to better health outcomes, where primary care practitioners are already educated on the diagnosis and management of mental health disorders.

#### 3.1.3. Psychotherapy: Cultural Adaptations for Refugee Mental Health

Culture influences how mental health conditions are generated, experienced, and treated. This highlights the need for an effective and inexpensive process that is both easy to administer and culturally sensitive. Cultural adaptation is the “systematic modification of evidence-based treatment to account for language, culture, and context that is consistent with the client’s cultural patterns, meanings, and values” [38]. In addition, culture may influence the acceptability of psychotherapy interventions. For example, cultures that value oral tradition and history telling may find narrative approaches such as NET socially acceptable, thereby, potentially countering the stigma associated with traditional mental health services [36]. Similarly, investigators suggested that CBT was particularly useful for Southeast Asian refugees owing to the similarity of Buddhist principles to core aspects of CBT (e.g., mindfulness) [40] and the ability to incorporate culturally appropriate visualization, such as a lotus bloom [41]. Furthermore, practitioners of CETA tailored skills to the individual and familial needs of their clients, as well as to the cultural needs of the community, by using culturally relevant folktales, personal anecdotes, and local expressions or adages to convey key principles [29]. Cultural modifications also included building on existing strengths (e.g., support of family and community) and existing coping strategies (e.g., meditation, singing songs, having tea with friends) to increase daily functioning [29].

#### 3.1.4. Psychotherapy: Intensity

Primary health care typically has a limited early focus on mental health. The majority of our included studies achieved positive expected outcomes by observing the recommended intensity of psychotherapies according to the treatment manuals. However, three trials adopted a more brief version of psychotherapies [36,42,48]. Two studies on NET conducted three treatment sessions of 90 to 120 min duration [36,48]. The standard version of NET is a median of 9–10 treatment sessions with 120 min duration [46]. One study provided 6 biweekly sessions of IPT compared to the traditional version of 12–16 individual weekly sessions [42]. In studies offering brief interventions, weaker symptom improvement was due to the limited dose of treatment sessions, coupled with the relatively severe and often long-standing trauma symptoms in the sample [36]. In contrast, the high intensity (16–20 sessions, 120 min in length) of CBT and exposure therapy was reported as a factor contributing to these psychotherapies’ positive significant results [49]. If a considerably lesser amount of, and shorter sessions, had been used, the clinical impression is that both exposure therapy and CBT would not have resulted in significant improvements in the patients [49]. In cases where the refugee patient does not speak the local language, the presence of an interpreter also has implications for psychotherapy intensity: Not only must the patient trust the interpreter enough to allow them to participate in the treatment, but the treatment itself must proceed at a considerably slower pace and be long enough so that adequate time is allotted to all aspects of the treatment [49]. Indeed, variable outcomes may reflect the limitations of short-term psychosocial interventions [48].

### 3.2. Theories of Why Community-Based Mental Health Services for Refugees Do Not Work

#### 3.2.1. Clinician: Diverse Socio-Cultural Differences

Social and cultural factors influence how patients from a given culture express and manifest their symptoms, their style of coping, their family and community support, and their willingness to seek treatment. Importantly, the current Diagnostic and Statistical Manual of Mental Disorders (DSM) PTSD criteria may not represent the full spectrum of response to trauma across different cultural contexts, and culture-specific reactions to trauma need to be elucidated. Thus, there is not always content equivalence in the symptomatology of trauma-related disorders in different cultural groups [40]. As such, clinicians should consider socio-cultural factors when selecting interventions. For example, a study on Stress Inoculation Training (SIT) found no reduction in PTSD symptoms among asylum seekers in Germany [35]. This may be due to differences in participant education and other sociocultural factors. SIT may be more successful with more highly educated patients because it requires an understanding of abstract concepts (i.e., the distinction between thoughts, feelings, and behavior). Moreover, it is possible that some of the treatment components of SIT were outside of the cultural norms of the participants because it has been developed according to the Western understanding of human experiences and behavior [35].

Socio-cultural factors, such as gender roles, may influence the selection of the clinician. In the literature, female patients withdrew from interventions because they did not want a male therapist [49] or because their husbands “forbade them to continue” [42]. Evidence indicated that mixed gender pairing between patient and clinician may require the presence of another clinician during the encounter [30], highlighting the need for gender-sensitive approaches to care.

#### 3.2.2. Clinician: (Lack of) Mentorship and Advocacy

Mentorship is an important aspect within medicine and among psychotherapists. Physicians often work in a team-based approach and turn to colleagues for mentorship, consult on clinical cases, and share knowledge from clinical experience. High-quality training, supervision, and emotional and technical support are paramount to the success of psychotherapy implementation in primary health care settings (see Supplementary File S6). For example, the CETA trial employed levels of outside (i.e., USA-based) monitoring and supervision that may not be feasible in other settings like low resource clinics. High levels of mentoring were necessary to ensure that CETA was delivered with fidelity by newly trained providers [29]. Notably, this intervention also adopted a task-shifting approach; however, it was recommended that counsellors and supervisors meet weekly to go over the details of each case [30], a level of mentorship that may not be feasible within every practice.

Further, psychotherapy sessions have the potential to be an avenue for advocacy, which is rarely recognized. For example, at the end of NET treatment, patients obtain a written version of their narrative [52]. This hard copy of their biography can then be used for advocacy, including court proceedings and the refugee determination process as well as a testimony to the atrocities lived by the refugee. Many patients submitted their narratives to the court of human rights as proof of the violations of human rights occurring in their countries [46,48]. This is an opportunity for patients to feel fulfilled by their therapeutic experience and is also an opportunity for primary care clinicians in high-income countries to contribute to advocacy for refugees.

#### 3.2.3. Psychotherapy: Risk for Vicarious Trauma

Vicarious trauma is the secondary trauma encountered by mental health clinicians when working with patients with a history of trauma or PTSD. It is a process of internalizing the patient’s experiences through empathetic engagement and can lead to transformations within the clinician resulting in symptoms similar to that of the patient [53]. Given this risk, some psychotherapies may be better suited for non-professionals. For example, IPT does not involve detailed recounts of traumatic memories, thus reducing the risk of vicarious trauma compared to exposure therapies. In this respect, IPT may have a broader safety margin for delivery by lay therapists and providers in post-conflict communities [42].

#### 3.2.4. Patient: Insecure Asylum Status

Precarious political status limits access to basic services, including primary care and mental health services. Insecure asylum status can lead to an ongoing sense of insecurity and injustice, further exacerbating symptoms of PTSD for patients with experience of trauma. Such insecurities can lead to fear and limited engagement with mental health interventions. For example, in a study examining the effectiveness of NET vs SIT (a form of Stress Management (SM)), the majority of patients were in a continuous state of fear of being deported and one participant went into hiding for fear of deportation [35]. Under such conditions of “continuous trauma”, SIT may not be an effective treatment. Transfer of the newly taught stress-reducing exercises to everyday stressful situations may not work where there is a serious ongoing threat [35]. In a trial of CETA, few mental health services were available to Burmese refugees in Thailand except counselling at the Burmese-run Mae Tao Clinic. Many Burmese refugees were reluctant to go to the clinic or other places due to fear of deportation by Thai authorities [29]. One study associated high patient dropout rate with forced repatriation and migration resettlement programs [47]. Finally, there is a possibility that being a refugee in a country that one perceives as responsible for one’s victimization generates conflicted feelings and resentment that might exacerbate the original trauma and hinder successful emotional processing of it [36].

## 4. Discussion

Mental health interventions are often embedded in social systems, and how they work is shaped by this socio-cultural context [54]. To our knowledge, this is the first realist-informed review to explore contextual factors that influence psychotherapy for refugees. Our findings recognize the integral role that culture plays in the presentation, identification, and treatment of trauma. Mental health consequences of trauma may be effectively addressed by a primary care program within the “medical home” and using a trauma-informed approach. A program may deliver a culturally adapted psychotherapy with sufficient intensity in a migrant-friendly environment. Meeting such program requirements necessitates a commitment to building clinician capacity, interdisciplinary human resource allocation, mentorship, and advocacy. Given that primary care clinicians are often the first point of contact for refugee patients, we argue that this program is best placed within primary care.

Several systematic reviews have examined the effectiveness of psychotherapies for refugees. NET and CBT have the most robust evidence base for refugee populations [55,56,57]. Interestingly, effect sizes for the effectiveness of NET are substantially larger when delivered by refugee community counselors as opposed to clinical practitioners [58]. However, the mechanisms by which this larger effect occurs remain unknown. Our realist synthesis suggests that a shared lived experience of trauma or shared culture between practitioners and patients may reduce stigma and improve understanding. Existing reviews also report that asylum seekers and displaced persons face the uncertainty of protection and fear of return to danger [59], and that insecure residence status increases the risk of mental health problems. As such, empirical evidence suggests that programs that are effective for PTSD in the general population may not completely overlap with those that are appropriate for PTSD in asylum seekers and refugees [57]. While trauma-focused therapies for refugees reduce symptoms of PTSD and depression, more research is needed on the role of cultural factors and programs in the treatment of refugee populations [60].

Clinicians have advocated for shifts away from the medicalization of mental health and towards cultural competence in trauma care [61]. Some authors have argued that the DSM V PTSD criteria do not align with presentations and may pathologize normal emotions, including justified anger, and impede the natural recovery of communities and true integration into new societies [56,62]. Furthermore, trauma-focused approaches may not adequately address the needs of refugees with psychosocial stressors such as poverty, changes in support networks due to loss and migration, unsafe living conditions, and poor access to basic resources [60]. Psychotherapies within the context of an interdisciplinary primary care program involving social workers, nurses, and cultural interpreters as well as encompassing different elements of therapy, such as CETA, may be able to target mental health conditions while simultaneously addressing some of the comorbid psychosocial stressors [29]. Increasingly, primary health care is being viewed as the gateway to support the social determinants of health [63,64,65]. Indeed, there is a gap that primary care clinicians can fill to embed global mental health into routine primary health care.

### 4.1. Strengths and Limitations

Our realist-informed review followed a transparent and systematic methodology [16,66]. This approach allowed us to develop an understanding of what works, for whom, in what circumstances and why. Rather than controlling for real-life events, our realist synthesis provided a framework for working with and untangling the complexity of real-life implementation of psychotherapies for refugees. This allowed for an equal focus on what works, as much as what does not work, in an attempt to learn from failures and maximize learning across policy, disciplinary and organizational boundaries.

Restriction of our inclusion criteria to randomized controlled trials allowed us to assess effectiveness but does not report on rich contextual qualitative factors. Further, no studies were conducted in primary health care, and the majority of studies poorly described their settings of care. We also focused on psychotherapies for individuals only. Primary health care systems are typically oriented to provide services for individuals. Programs must consider the impact of trauma on the whole family as many refugee families share a collective trauma experience, and their healing journey must also be a shared process. Finally, although publication in a language other than English was not an exclusion criterion for studies, we did not search dedicated non-English language databases. Work with refugee populations is relevant across cultures, and we recognize additional evidence in this topic area may exist.

### 4.2. Implications for Research and Practice

As refugees settle and seek community-based care, they encounter barriers including poor access to mental health specialists. Primary care clinicians are well positioned to screen, diagnose, and manage refugees with complex mental health conditions. At the same time, primary health care clinicians are developing innovative interdisciplinary programs, for example pain management, diabetes, and palliative care. Mental health care of refugees is indeed complex and deserves interdisciplinary programs that can reduce stigma, improve access and adherence, build interdisciplinary care, and develop psychotherapy skills [67,68,69,70]. Links to cultural psychologists and psychiatrists will also improve diagnostics and treatment plans.

Task shifting [71], program composition and management, and refugee community engagement will all need research. Referral to specialized psychological services outside primary care, when access to interpreters and cultural brokers is not guaranteed, will also require community-based research. Community engagement research is needed to study adherence, follow-up, and stigma that can lead to poor mental health outcomes.

## 5. Conclusions

Our review identified practice, clinician, psychotherapy, and patient factors that contribute to the success and failure of psychotherapy for refugees. Several psychotherapies reduced symptoms and led to improved function and well-being in refugees. Task-shifting to primary care may help ease wait times, reduce mental health stigma, and build a community based “medical home”. However, the implementation of psychotherapy programs in primary care faces numerous logistic, clinical, and remuneration challenges. Empowering primary-care clinicians with the necessary skills, language, and program supports and knowledge may improve refugee care and reduce unnecessary specialist referrals.

## Figures and Tables

**Table 1:** Principles of trauma-informed programs and care (Adapted from Purkey et al. [4]).

Principle	Applying the Principle
Trauma awareness and acknowledgment	Be aware of the prevalence and effect of trauma on substance use, physical and mental health, and ensure that all staff members understand how trauma affects life’s experiences Recognize the effects of violence and abuse on a patient’s development and coping strategies Recognize the pervasiveness and long-term effects of violence and abuse
Safety and trustworthiness	Help patients feel they are in a safe place Recognize the need for physical and emotional safety Avoid interventions that might trigger or re-traumatize a patient Design services that maximize access and participation by trauma survivors (including flexibility in scheduling) Promote humility and cultural competence for all who interact with patients
Choice, control, and collaboration	Include patients in decisions affecting treatment Develop a collaborative relationship Involve service users when designing and evaluating services
Strengths-based and skill-building care	Support a patient’s empowerment Highlight a patient’s strengths and resilience rather than focusing on symptoms and pathology
Cultural, historical, and gender issues	Incorporate processes that are sensitive to a patient’s culture, ethnicity, and personal and social identity, as well as to his or her experience of trauma associated with group marginalization

**Table 2:** Realist-informed analysis.

Analysis Step	Description
1	Organization of extracted data into evidence tables, supplemented by information from training manuals when appropriate;
2	Theming by individual reviewers;
3	Comparison of reviewers’ themes for a specific article and formulation of chains of inference from the identified themes;
4	Linking of the chains of inference and tracking and linking of articles;
5	Hypothesis formulation (context, mechanism, and outcome configurations).
**Key definitions**	For the purpose of this review, we defined “context” as *the interrelated conditions in which a psychotherapy is delivered*; “mechanism” as *a combination of resources offered by the psychotherapy and stakeholders’ reasoning in response* [24]; and “outcome” as *short, medium and long term changes, intended and unintended, resulting from psychotherapy* [25].

**Table 3:** Characteristics of Included Studies.

Study ID	Related Training Materials	Study Design	Study Objective	Study Setting, Location, and Duration	Participant Characteristics	Intervention	Comparison	Language	Provider, Presence of Interpreter
Adenauer et al. 2011 [26]	Schauer, Neuner and Elbert, 2017 [27] Elbert, Schauer and Neuner, 2015 [28]	Randomized control trial	To examine whether narrative exposure therapy (NET) causes changes in affective stimulus processing in patients with chronic PTSD	The Psychological Research and Outpatient Clinic for Refugees at the University of Konstanz. Konstanz, Germany. Follow-up: 4 months	Participants were refugees and asylum seekers with a history of organized violence or persecution and current PTSD diagnosis Total sample size n = 34 Treatment n = 16 Control n = 18	Narrative Exposure Therapy (NET) was used as a variant of trauma-focused CBT. NET is a manualized short-term approach that has been adapted to meet the needs of traumatized survivors of war and torture. Average number of sessions: 12 Average length of sessions: 108 min (SD = 17) Frequency of sessions: weekly or biweekly	Participants in the control group were waitlisted.	Not reported	Clinical psychologists of the University of Konstanz with expertise in PTSD and NET carried out the treatment according to the manual, with the help of a translator if necessary.
=Bolton et al. 2014 [29]	Murray et al. 2013 [30]	Randomized control trial	To test a transdiagnostic treatment developed for comorbid presentations of depression, anxiety, and trauma symptoms among trauma survivors in a low-resource setting	Burmese-run MTC 5 km from Myanmar. Mae Sot, Northwest Thailand. Follow-up: 4 months	Participants were Burmese individuals at least 18 years of age who have witnessed or experienced a traumatic event and suffer from moderate to severe depression and/or PTSS Total sample size n = 347 Treatment n = 182 Control n = 165	CETA CBT is a transdiagnostic treatment approach developed for delivery by lay counselors in low resource settings with few mental health professionals. CETA was designed to treat symptoms of common mental health disorders including depression, PTS, and anxiety. Average number of sessions: 9.7 (7 to 13) Average length of sessions: 1 h Frequency of sessions: weekly	Participants in the control group were waitlisted	Burmese	Counselors and supervisors were staff at one of three local service organizations. All were Burmese refugees, or members of the Burmese community in Mae Sot, and shared many cultural, religious, and political experiences with their clients.
Carlsson et al. 2018 [31]	Vindbjerg et al. 2014 [32] Lehrer et al. 2008 [33] Anderson et al. 2011 [34]	Randomized control trial	To compare the effectiveness of CBT with a focus on stress management (SM) or cognitive restructuring (CR) in a clinical sample of trauma-affected refugees	The Competence Centre for Transcultural Psychiatry (CTP); an outpatient clinic. Copenhagen, Denmark Follow-up: 6–7 months	Participants were refugees or family unified with refugees who have obtained asylum in Denmark and have trauma-related mental health problems Total sample size n = 126 SM n = 62 CR n = 64	Stress management (SM): The primary goal of the therapy is to help patients acquire and consolidate a number of coping skills. Thus, the sessions focus on learning and applying new coping skills. The SM manual used in this study included the following techniques: (1) relaxation, (2) attention diversion and (3) behavioral activation. Average number of sessions: 16 of SM with 10 sessions with a doctor Average length of sessions: 45–60 min Frequency of sessions: Not reported Length of program: 6–7 months	Cognitive restructuring (CR): The CR manual consisted mainly of psychoeducation and cognitive restructuring of negative thoughts resulting from traumatic experiences and exposure. Each theme consisted of psychoeducation, suggestions for interventions as well as suggestions for homework assignments.	Not reported All self-administered questionnaires were available in 5 languages: Arabic, Bosnian, Danish, English, and Farsi	Participants in both groups were offered sessions with a medical doctor and sessions of psychotherapy with a psychologist. All patients in need of an interpreter received this assistance and, if possible, the same interpreter was used throughout the treatment.
Hensel-Dittman et al. 2011 [35]	Schauer, Neuner and Elbert, 2017 [27] Elbert, Schauer and Neuner, 2015 [28] Lehrer et al. 2008 [33]	Randomized control trial	To compare the outcome of 2 active treatments for posttraumatic stress disorder (PTSD) as a consequence of war and torture: narrative exposure therapy (NET) and stress inoculation training (SIT)	The Research and Outpatient Clinic for Refugees, a unit operated jointly by the University of Konstanz and the NGO Vivo Konstanz, Germany Follow-up: 12 months	Participants were asylum seekers who had fled their country of origin after experiencing organized violence and had a current PTSD diagnosis Total sample size N = 28 NET n = 15 SIT n = 13	In NET, the participant constructs a detailed chronological account of his or her own biography in cooperation with the therapist. Empathic understanding, active listening, congruency, and unconditional positive regard are key components of the therapist’s behavior. Average number of sessions: 10 Average length of sessions: 90 min Frequency of sessions: weekly or biweekly	SIT is a cognitive behavioral semi- structured program aimed at enhancing the patient’s ability to cope with stress. Techniques applied in SIT are training in breathing techniques, relaxation training, cognitive restructuring, thought stopping, guided self-dialog, covert modeling, and role play.	Not reported	Therapists were trained staff from the Research and Outpatient Clinic for Refugees. Treatment was usually carried out by 1 therapist, with 1 trainee therapist observing and assisting in the sessions.
Hijazi et al. 2014 [36]	Schauer, Neuner and Elbert, 2017 [27] Elbert, Schauer and Neuner, 2015 [28]	Randomized control trial	To test the effects of an adapted brief Narrative Exposure Therapy in a sample of traumatized Iraqi refugees	At the participant’s preferred location (typically the home but sometimes a church or community center) Southeast Michigan, United States Follow-up: 4 months	Participants were Arabic-speaking adult Iraqi refugees who had resettled in southeast Michigan and had been exposed to a violent or traumatic event and were bothered by it. Total sample size n = 63 NET n = 41 Control n = 22	Brief NET: Three sessions, lasting 60–90 min each and included psychoeducation. The participant then constructed a chronological narrative of his or her life, starting with highlights of childhood and then focusing on traumatic experiences during adulthood. At these trauma points, the therapist encouraged the participant to describe sensory, cognitive, and emotional experiences. Average number of sessions: 3 Average length of sessions: 60–90 min Frequency of sessions: weekly	Participants in the control group were waitlisted.	Arabic	Therapists received training and weekly supervision by a licensed psychologist with expertise in exposure therapies.
Hinton et al. 2009 [37]	Hinton and Patel, 2017 [38] Hinton and Jalal, 2014 [39]	Repeated- measures randomized control trial	To examine the effect of a culturally sensitive CBT for traumatized Cambodian refugees with PTSD and comorbid orthostatic panic attacks.	A community-based outpatient clinic City not reported, United States Follow-up: 12 and 24 weeks	Participants were Cambodian patients who were considered to have pharmacology-resistant PTSD with comorbid orthostatic panic. Total sample size n = 24 Initial treatment n = 12 Delayed treatment n = 12	CBT was offered across 12 weekly sessions and emphasized information about a cognitive-behavioral model of PTSD and panic disorder, muscle relaxation and diaphragmatic breathing, guided imagery and mindfulness training. The therapy emphasized various techniques to promote emotional regulation. Average number of sessions: 12 Average length of sessions: Not reported Frequency of sessions: weekly	Treatment as usual: Supportive psychotherapy, which consisted of a meeting with a social worker every 2 weeks, and medications, which consisted in all cases of a combination of an SSRI (in most cases, paroxetine) and the benzodiazepine, clonazepam. After the initial treatment was finished, the delayed treatment group were given CBT as well.	CBT was provided in Cambodian Measures were translated to Khmer and then to English	The first co-author (Devon Hinton, Psychiatrist) who is fluent in Cambodian delivered or co-led the CBT treatment.
Hinton et al. 2005 [40]	Hinton and Patel, 2017 [38] Hinton and Jalal, 2014 [39]	Repeated- measures randomized control trial	To examine the therapeutic efficacy of a culturally adapted third generation cognitive-behavior therapy for Cambodian refugees with treatment-resistant posttraumatic stress disorder (PTSD) and comorbid panic attacks	A community-based outpatient clinic City unidentified, United States Follow-up: 12, 24, and 36 weeks	Participants were Cambodian patients who were treatment resistant; that is, still meeting PTSD criteria despite receiving supportive counseling and SSRI. Total sample size n = 40 Initial treatment n = 20 Delayed treatment n = 20	Individual CBT was offered across 12 weekly sessions, providing information about the nature of PTSD and Panic Disorder, muscle relaxation and diaphragmatic breathing procedures, performing a culturally appropriate visualization, providing an emotional-processing protocol, etc. Average number of sessions: 12 Average length of sessions: Not reported Frequency of sessions: weekly	Treatment as usual: All patients continued supportive psychotherapy, which consisted of a meeting with a social worker every 2 weeks, and medications, which consisted in all cases of a combination of an SSRI and the benzodiazepine clonazepam. After the initial treatment was finished, the delayed treatment group were given CBT as well.	CBT was provided in Cambodian Measures were translated to Khmer and then to English	The first co-author (Devon Hinton, Psychiatrist), who is fluent in Cambodian, conducted the CBT sessions.
Hinton et al. 2004 [41]	Hinton and Patel, 2017 [38] Hinton and Jalal, 2014 [39]	Repeated- measures randomized control trial	To examine the feasibility, acceptability, and therapeutic efficacy of a culturally adapted cognitive behavior therapy (CBT)	Two community-based outpatient clinics that provided specialized services to non-English speaking Cambodian and Vietnamese refugees City not reported, United States Follow-up: 11 and 22 weeks	Participants were Vietnamese patients (practicing Buddhists) who met PTSD criteria despite at least 1 year of SSRI and supportive counseling. Total sample size n = 12 Immediate treatment n = 6 Delayed treatment n = 6	Individual CBT was offered across 11 weekly sessions, providing information about the nature of PTSD and panic disorders, training muscle relaxation and diaphragmatic breathing procedures, culturally appropriate visualization, cognitive restructuring of fear networks, conducting interoceptive exposure, etc. Average number of sessions: 11 Average length of sessions: Not reported Frequency of sessions: weekly	Participants in the delayed group received CBT after the initial treatment group finished their treatment.	Vietnamese	The first author (Devon Hinton, Psychiatrist) led the CBT sessions. Vietnamese social workers and staff provided translation and cultural consultation.
Meffert et al. 2014 [42]	Stuart, 2006 [43] Stuart and Robinson, 2012 [44] Weissman, Markowitz and Lerman, 2007 [45]	Randomized control trial	To examine the impact of interpersonal psychotherapy (IPT) on Sudanese refugees living in Cairo, Egypt, who had symptoms of PTSD	Screening and therapy were conducted at the offices of Ma’an Organization; a Sudanese founded and run NGO Cairo, Egypt Follow-up: 3 weeks	Participants were Sudanese refugees living in Cairo, Egypt who had difficulties with their mental health and relationships but without severe thought or mood disorder symptoms. Total sample size n = 22 IPT n = 13 Control n = 9	6 bi-weekly sessions of IPT; a brief and highly structured manual-based psychotherapy. IPT aims to intervene specifically in current social functioning with consequent benefits for symptom experience. IPT does not focus on retelling of past traumatic experiences; rather, the goal is to change current relationships to improve mood symptoms. Average number of sessions: 6 Average length of sessions: Not reported Frequency of sessions: twice per week	Individuals assigned to the waitlist condition were offered IPT treatment at the conclusion of the therapy in the intervention group.	Sudanese	Five members of the Sudanese community without prior mental health training were trained to deliver IPT. The translation team consisted of four men from Sudan. Two were certified translators and interpreters, one worked as a full-time interpreter, and one was a community leader in Cairo.
Neuner et al. 2010 [46]	Schauer, Neuner and Elbert, 2017 [27] Elbert, Schauer and Neuner, 2015 [28]	Randomized control trial	To examine the efficacy of trauma-focused treatment mainly Narrative Exposure Therapy among asylum seekers with PTSD	The Psychological Research and Outpatient Clinic for Refugees at the University of Konstanz Konstanz, Germany Follow-up: 6 months	Participants were asylum seekers with a 3 months temporary leave to remain, who suffered a history of victimization by organized violence, and fulfilled the DSM-IV criteria for PTSD. Total sample size n = 32 NET n = 16 TAU n = 16	NET consisted of a median of nine treatment sessions with an average duration of 120 min. Sessions were scheduled on a weekly or biweekly basis. Treatment was terminated at the therapist’s discretion as soon as the patient could, according to clinical judgment, talk about his or her traumatic experiences in detail without avoidance, memory gaps, or being emotionally overwhelmed. Average number of sessions: 9 (SD 3.77) Average length of sessions: 120 min Frequency of sessions: weekly or biweekly	Patients who were randomized to the treatment as usual condition were encouraged to continue their current treatment or were referred to institutions of public mental health care.	German	NET treatment was carried out by therapists who were doctoral- level psychologists and graduate students with extensive training in NET from the University of Konstanz. Treatment with interpreters was offered for patients who were not fluent in German.
Neuner et al. 2008 [47]	Schauer, Neuner and Elbert, 2017 [27] Elbert, Schauer and Neuner, 2015 [28]	Randomized control trial	To examine whether trained lay counselors can carry out effective treatment of posttraumatic stress disorder (PTSD) in a refugee settlement	The Nakivale refugee settlement, one of eight official refugee camps in Uganda Isingiro District, Southwest Uganda Follow-up: 9 months	Participants were Rwandan and Somalian refugees who were diagnosed with PTSD. Total sample size n = 277 NET n = 111 TC n = 111 MG n = 55	Narrative Exposure Therapy: Six sessions (usually two sessions per week); where the participant constructs a detailed chronological account of his own biography in cooperation with the counselor. During the discussion of traumatic experiences, the counselor asks for current emotional, physiological, cognitive, and behavioral reactions and probes for respective observations. The participant is encouraged to relive these emotions while reporting the events. Average number of sessions: 6 Average length of sessions: 1 or 2 h Frequency of sessions: two sessions weekly	Trauma Counselling: Six sessions (usually two sessions per week), a combination of a variety of treatment and counseling methods that could be applied for different cases at the discretion of the Therapist. A main principle of TC was to relate current problems to past traumatic experiences.	Somali and Rwandan	Lay counselors: nine refugees (five women, four men) from the community were trained via a 6-week course in general counseling skills (e.g., active listening, empathy, verbalization, emotional processing, etc.) as well as specific abilities and methods that were needed for both treatment approaches. The trainers were five postdoctoral- and doctoral-level personnel
Neuner et al. 2004 [48]	Schauer, Neuner and Elbert, 2017 [27] Elbert, Schauer and Neuner, 2015 [28]	Randomized control trial	To evaluate the efficacy of narrative exposure therapy, supportive counseling, and psychoeducation for treating PTSD	The Imvepi settlement in northern Uganda Arua District, Northwestern Uganda Follow-up: 12 months	Participants were Sudanese refugees living in a Ugandan refugee settlement who met the DSM-IV criteria for PTSD Total sample size n = 43 NET n = 17 Counseling n = 14 Psychoeducation n = 12	In narrative exposure therapy, the first session always included psychoeducation about the nature and prevalence of PTSD symptoms. The participant constructs a detailed chronological account of his own biography in cooperation with the therapist. A special focus of the therapy is on the transformation of the generally fragmented report of traumatic experiences into a coherent narrative. Average number of sessions: 4 Average length of sessions: 90 to 120 min Frequency of sessions: Within 2 weeks	In supportive counseling, the first session always included psychoeducation about the nature and prevalence of PTSD symptoms. The main goal of supportive counseling was to explore and strengthen the participants’ individual, social, and cultural resources. The focus of them treatment was on current interpersonal problems, personal decisions, and plans and hopes for the future. For participants in the psychoeducation group, no further treatment was offered.	All instruments were translated into the Arabic dialect spoken by the refugees in Imvepi (Juba-Arabic).	Treatment was carried out by three female and two male therapists from the University of Konstanz and the aid organization Vivo with the help of interpreters.
Paunovic et al. 2001 [49]	Hinton and Patel, 2017 [38] Hinton and Jalal, 2014 [39]	Randomized control trial	To investigate the efficacy of cognitive-behavior therapy CBT and exposure therapy in the treatment of post-traumatic stress disorder PTSD in refugees	Setting not identified. Referrals happened in psychiatric units and the Center for tortured and traumatized refugees at the Karolinska hospital. Stockholm, Sweden Follow-up: 6 months	Participants were refugee patients who met the DSM-IV criteria for PTSD, can speak the Swedish language, and have a lasting Swedish residence permit. Total sample size n = 12 CBT n = 6 ET n = 6	Cognitive Behavioral Therapy CBT included a flexible combination of exposure, cognitive therapy, and controlled breathing. Controlled breathing was used in order to help the patient control the irregular breathing. Cognitive therapy was used in order to teach the patient to decatastrophize his/her interpretations of intrusive recollections. Average number of sessions: 16–20 sessions Average length of sessions: 60–120 min Frequency of sessions: weekly	Exposure Therapy (ET): Patients were gradually confronted with anxiety-provoking trauma-related images and situations with the help of the therapist. Each step was completed when the patient successfully habituated to the trauma cues within and/or between the sessions. Each exposure lasted 20–60 min.	Swedish	Treatment was conducted by the first author, a doctoral student in clinical psychology. The therapist was familiar with E and CBT in the treatment of PTSD for 3 years.
Sternmark et al. 2013 [50]; Halvorsen et al. 2014 [51]	Schauer, Neuner and Elbert, 2017 [27] Elbert, Schauer and Neuner, 2015 [28]	Randomized control trial	To compare Narrative Exposure Therapy NET to treatment as usual in 11 general psychiatric health care units in Norway	Eleven centers in the general psychiatric services City not reported, Norway Follow up: 6 months	Participants were refugees and asylum seekers fulfilling the DSM-IV criteria for PTSD who had been referred to treatment in the general psychiatric services in Mid-Norway. Total sample size n = 81 NET n = 51 TAU n = 30	In the NET condition, the patients are assisted to construct a chronological narrative of their life stories with special emphasis in the traumatic experiences. Active listening and empathic understanding are key elements in the therapist’s efforts to transform fragmented reports of traumas into coherent narratives. 10 sessions of 90 min duration. Average number of sessions: 10 Average length of sessions: 90 min Frequency of sessions: weekly	In the treatment as usual (TAU) condition, the therapists were instructed to use any intervention they normally would use, except for the steps specific to NET. TAU mainly consisted of help with such as sleep problems, depressive symptoms, problems related to asylum status, and other practical matters.	Norwegian or English	Twenty-four experienced mental health professionals including psychologists, psychiatrist, psychiatric nurses, occupational therapists, drama therapists, and clinical social workers. If the patients were not reasonably fluent in Norwegian or English, certified translators assisted both in the assessments and in the treatments.

## Supplementary Materials

The following are available online at https://www.mdpi.com/1660-4601/17/13/4618/s1. Table S1, RAMESES Publication Standards: Realist Synthesis; Table S2, Inclusion and exclusion criteria; Table S3, Search strategy; File S4, Critical appraisal; Table S5, Effectiveness of psychotherapies; Table S6, Key elements and provider training.
