# Supplementary material for: Understanding Supporting and Hindering Factors in Community-Based Psychotherapy for Refugees: A Realist-Informed Systematic Review
Source: Int J Environ Res Public Health. 2020 Jun 27;17(13):4618. doi: 10.3390/ijerph17134618 (PMC7369747; doi:10.3390/ijerph17134618)
Supplement: Supplementary file 1 [file ijerph-17-04618-s001.zip › S6_File Key elements and provider training.docx]

**Supplementary File S6: Key elements and provider training**

| **Psychotherapy** | **Key elements** | **Provider training** |
| --- | --- | --- |
| CBT | The focus of CBT is problem-oriented, with an emphasis on the present. It aims to change how a person thinks (‘cognitive’) and what they do (‘behaviour’). CBT therefore uses both cognitive and behavioural techniques.  Cognitive techniques: A key cognitive concept in CBT is ‘guided discovery’ which involves trying to understand the patient’s view of things and help them expand their thinking to become aware of their underlying assumptions, and discover alternative perspectives and solutions for themselves. To target maladaptive core beliefs, the patient can be asked to keep a positive observation data log. To target dysfunctional assumptions, the patient can be asked to provide evidence that supports/does not support their assumptions. Thought records are used to make a patient aware of their negative automatic thoughts, distinguish thoughts from facts, and see how they impact upon their mood.  Behavioural techniques: Activity scheduling and graded task assignment aim to enhance functioning and systematically increase pleasurable or productive experiences. | Reading and lectures/talks were most frequently rated as effective strategies for learning declarative knowledge and conceptual knowledge/skills, but relatively poor strategies for learning procedural skills, particularly in the interpersonal domain.  Modelling was a highly rated strategy for both declarative and procedural learning, and for conceptual and technical  knowledge/skills acquisition.  Role-play was most strongly associated with procedural skills learning, particularly for technical and interpersonal skills.  Reflective practice and self experiential work showed similar patterns of perceived effectiveness. Both were perceived to be effective in enhancing the procedural and reflective systems, particularly for the learning  of interpersonal skills. |
| CETA | The Common Elements Treatment Approach CETA is a transdiagnostic intervention for adults in low-resource settings presenting with mood and/ or anxiety problems. Lay counselors are trained on the following elements:   1. Encouraging engagement with special attention on perceptual and concrete obstacles 2. An introduction of psychoeducation where program duration, content and expectations are explained and symptoms/ problems are normalized and validated 3. Anxiety management strategies to improve physiological tension 4. Behavioural activation by identifying and engaging in pleasurable, mood boosting activities 5. Cognitive restructuring of the association between thoughts, feelings, and behaviour 6. Gradual imaginal exposure to feared and avoided memories and gradual desensitization 7. In vivo exposure by facing innocuous triggers in the client’s environment 8. Safety assessment and developing a focused safety plan 9. Motivational interviewing to help change substance use behaviours. | Each training component has a manual section and “steps sheets” that include both goals and example wording for these goals. The sheets were designed for use when practicing as well as during the sessions.  Local supervisors and counselors are taught strategies for identifying the primary problem areas for each client (traumatic stress and depression, predominantly depression). This process is data-driven, with decisions based on client responses on locally validated assessment measures, clinical presentations, and discussion with local supervisors.  Counselors are taught to use the weekly symptom monitoring to inform dose for each component and any areas of “interference” where additional elements might be needed to be added.  During training, brief case vignettes are presented, some with assessment results, to allow counselors to practice element selection, sequencing, and dosing.  Counselors work in small groups to select and sequence cards of the CETA elements for each case vignette |
| IPT | IPT focuses specifically on interpersonal relationships with the goal of helping patients to either improve their interpersonal relationships or change their expectations about them.  1. Assessment: To evaluate the suitability of the patient for IPT as well as the suitability of IPT for the patient  2. Initial sessions that:   1. Fully assess psychiatric and interpersonal problems 2. Develop an Interpersonal Formulation 3. List the patient’s key current relationships and the problems associated with them 4. Identify problem areas: disputes, role translations, grief and loss and interpersonal sensitivity 5. Explain the rationale and purpose of IPT and establish a Treatment Contract with the patient: emphasizing the focus of treatment, the time-frame, and the expectations for both the patient and therapist   3. Middle sessions to explore the patient’s expectations and perceptions of a specific interpersonal problem, brainstorm for possible solutions and implement them and use IPT specific techniques such as clarification, interpersonal incidents, encouragement of affect, problem solving  4. Conclusion of acute treatment: Where the provider review the patient’s progress and anticipate future problems  5. Maintenance treatment to establish a specific contract and monitor progress | Prospective IPT therapists should come from a counseling or medical background, attend a specified number of hours of didactic training in the theory and practice of IPT, and be supervised by a recognized expert in at least two IPT cases.  The current view of ISIPT is that clinical training in IPT should consist of at least:   1. General training in mental health, including the fields of medicine, psychology, social work, occupational therapy, or psychiatric nursing. 2. Attendance at a training program approved by ISIPT that includes at least 16–24 hours of didactic instruction. The course should highlight the theoretical and practical aspects of IPT, and should include opportunities for interaction via role plays and discussion of clinical material. 3. Supervision of at least one case with a supervisor recognized by the ISIPT, which includes audio or videotaped treatment sessions. |
| NET | The client, with the assistance of the provider, constructs a chronological narrative of her/his life story with a focus  on traumatic experiences. The patient is encouraged to relive these experiences while narrating, without losing the connection to the “here and now.” Using permanent reminders that the feelings and physiological responses result from activation of (hot) memories , the therapist links these mnemonic representations to episodic facts, that is, time and place (cold memory). The patient represents their memories using a “lifeline”, using flowers for positive memories and stones for negative memories. Together, the therapist and patient write a narrative and reflect on the meaning and content. During the final session the events constituting the individual’s life are reviewed as a contextualized and integrated narration  Several elements of NET have been identified as contributing to its efficacy that the clinician may wish to keep in mind:   1. Active chronological reconstruction of the autobiographical/episodic memory 2. Extended exposure to the “hot spots” and full activation of the fear memory in order to modify the emotional network (i.e., learning to separate the traumatic memory from the conditioned emotional response and understanding triggers as cues, which are just temporarily associated) through detailed narration and imagination of the traumatic event 3. Meaningful linkage and integration of physiological, sensory, cognitive, and emotional responses to one’s time, space, and life context (i.e., comprehension of the original context of acquisition and the reemergence of the conditioned responses in later life) 4. Cognitive reevaluation of behavior and patterns (i.e., cognitive distortions, automatic thoughts, beliefs, responses) as well as reinterpretation of the meaning content through reprocessing of negative, fearful, and traumatic events – completion and closure 5. Revisiting of positive life experiences for (mental) support and to adjust basic assumptions 6. Regaining of one’s dignity through satisfaction of the need for acknowledgement through the explicit human rights orientation of “testifying” | Training for NET is aimed at psychologists, physicians and psychotherapists or other psychosocial professions in public institutions and private practices, who work with traumatized patients or want to gain therapeutic expertise in the field of traumatic disorders.  Training in NET entails a 2-4 day course which covers the theoretical basics on NET as well as practical procedures. Training may require up to 3 weeks for lay counselors in areas of complex emergency or need. The workshops are facilitated by a team of scientists, clinical and field practitioners with expertise in clinical psychology, especially trauma-spectrum disorders, brain research as well as humanitarian aid. Trainings involve a combination of  lectures, exercises, role-plays and demonstrations, focused on the theoretical and practical skill training of the trauma practitioner. Case studies foster collective learning from real-life situations. The workshops create space for discussion and sharing of participants’ own experiences, ideas and knowledge. |
| SM | The concept of stress management is helpful because it is easy to communicate with a flexible manual focusing on current discrete problems rather than past traumas. SM techniques are categorized into three groupings:  1. Relaxation techniques that include breathing exercises, body relaxation and visualisation exercises, in which the patient is taught to visualize a secure, trauma free zone  2. Attention-diversion which entails the shifting away of thoughts, feelings or impulses from an unwanted or uncomfortable state.   1. Diversion is the conscious interruption of the ruminations, flashbacks and burdensome feelings which shift bleak thoughts, feelings or impulses towards other things so the patient is brought into contact with, and becomes aware of present circumstances. 2. Thought-stop aims for a short and intense interruption of unwanted thoughts and rumination. 3. Outer focus is letting oneself get immersed in what happens around oneself and thus shifting one’s attention away from mental and bodily processes. 4. Emotion control techniques to help patients find concrete coping thoughts to be used in situations perceived difficult   3. Behavioural activation techniques to increase the patient’s cognitive and physical functioning. Techniques include visualization of conducting an activity a patient wishes to be able to do master in real life, activity planning to give patients control over their lives, and evening therapy where a patient writes down three positive experiences, incidents or thoughts they experienced during the day. | Providers interested in delivering SM to their patients typically complete a two-day course which course includes the theory of stress and its management. The course takes a multimodal cognitive-behavioural approach to stress management and is based on current research and practice. Some of the topics included are individual and organisational symptoms of stress, thinking errors and thinking skills, stress mapping, stability zones, relaxation techniques, biofeedback, pressure and stress, lifestyle management, physical outlets, management of the personal work environment, Type A behaviour, locus of control, time management, coping strategies at work and home. The objectives are to:   1. define stress and understand how it differs from pressure 2. have a working understanding of modern models of stress including the multimodal-transactional model, the cognitive ABCDE model and an organisational model 3. understand the relevant neurobiology and psychophysiology of stress 4. identify the main physical, psychological and behavioural symptoms of stress in self and others 5. examine primary, secondary and tertiary stress management interventions at the individual and organisational levels 6. recognise thinking errors and performance interfering thoughts and develop coaching, training or counselling thinking skills to help individuals modify these beliefs 7. develop a range of strategies and techniques to tackle stress, including Type A modification, relaxation skills, lifestyle management, biofeedback, stress mapping 8. understand Type A behaviour and Locus of Control constructs 9. recognise the organisational symptoms of stress and identify what strategies can be undertaken to prevent and manage stress at work |

CBT, Cognitive Behavioural Therapy; CETA, Common Elements Treatment Approach; IPT, Interpersonal Therapy; NET, Narrative Exposure Therapy; SM, Stress Management
