# Supplementary material for: Understanding Supporting and Hindering Factors in Community-Based Psychotherapy for Refugees: A Realist-Informed Systematic Review
Source: Int J Environ Res Public Health. 2020 Jun 27;17(13):4618. doi: 10.3390/ijerph17134618 (PMC7369747; doi:10.3390/ijerph17134618)
Supplement: Supplementary file 1 [file ijerph-17-04618-s001.zip › S5Table_Effectiveness of psychotherapies.docx]

**Supplementary File S5: Effectiveness of psychotherapies**

| **Psychotherapy** | **Study** | **Participant attrition** | **Anxiety** | **PTSD** | **Depression** | **Conclusions** |
| --- | --- | --- | --- | --- | --- | --- |
| CBT | Hinton 2004 | No participants were lost to follow-up | Anxiety Sensitivity Index  Immediate treatment  T1: 43.5 (9.1)  T2: 18.5 (6.4)  T3: 21.0 (8.0)  Delayed treatment  T1: 38.07 (7.4)  T2: 47.6 (7.2)  T3: 20.2 (4.9)  Group Effect Size (Cohen’s d) at T2 d=4.3  Hopkins Symptom Checklist-25 (HSCL-25), anxiety subscale  Immediate treatment  T1: 3.5 (0.5)  T2: 2.2 (0.5)  T3: 1.8 (0.6)  Delayed treatment  T1: 3.3 (0.6)  T2: 3.2 (0.4)  T3: 2.1 (0.4)  Group Effect Size (Cohen’s d) at T2 d = 2.2 | Harvard Trauma Questionnaire  Immediate treatment  T1: 3.3 (0.4)  T2: 1.7 (0.5)  T3: 1.8 (0.7)  Delayed treatment  T1: 3.1 (0.7)  T2: 3.3 (0.8)  T3: 2.0 (0.6)  Group Effect Size (Cohen’s d) at T2 d=2.5 | Hopkins Symptom Checklist-25, depression subscale  Immediate treatment  T1: 3.1 (0.7)  T2: 2.1 (0.5)  T3: 1.6 (0.6)  Delayed treatment  T1: 3.3 (0.9)  T2: 3.2 (0.16)  T3: 1.9 (0.4)  Group Effect Size (Cohen’s d) at T2 d = 2.0 | Between-group comparisons indicated significant improvements, with large effect sizes (Cohen’s d) for all outcome measures: Anxiety, depression and PTSD.  No subgroup data reported. |
|  | Hinton 2005 | No participants were lost to follow-up | Anxiety Sensitivity Index  Immediate treatment  T1: 3.08 (0.61)  T2: 1.65 (0.45)  T3: 1.86 (0.32)  T4: 1.98 (0.40)  Delayed treatment  T1: 3.27 (0.53)  T2: 3.19 (0.36)  T3: 1.84 (0.42)  T4:1.91 (0.49)  Group Effect Size (Cohen’s d) at T2  3.78 | Clinician-Administered PTSD Scale  Immediate treatment  T1: 74.85 (14.67)  T2: 39.25 (19.92)  T3: 41.30 (13.95)  T4: 44.59 (14.58)  Delayed treatment  T1: 75.91 (11.5)  T2: 73.05 (9.43)  T3: 45.05 (8.72)  T4: 43.56 (10.22)  Group Effect Size (Cohen’s d) at T2  2.17 | Symptom Checklist-90-R  Immediate treatment  T1: 2.92 (0.61)  T2: 1.72 (0.43)  T3: 1.77 (0.30)  T4:2.02 (0.78)  Delayed treatment  T1: 3.02 (0.51)  T2: 2.94 (0.45)  T3: 2.03 (0.41)  T4: 1.96 (0.89)  Group Effect Size (Cohen’s d) at T2  2.77 | Patients improved on all measures, including fear of anxiety-related somatic sensations (ASI), PTSD severity (CAPS), anxiety and depression-related  distress (SCL subscales). Between-group comparisons indicated benefit over the waitlist condition on the order of very large effect sizes according to Cohen’s standards.  No subgroup data reported. |
|  | Hinton 2009 | No participants were lost to follow up |  | Clinician‐Administered PTSD Scale (CAPS)  Immediate treatment  T1: 75.41 (13.47)  T2: 46.83 (17.17)  T3: 44.75 (14.85)  Delayed treatment  T1: 77.25 (11.47)  T2: 74.25 (9.43)  T3: 45.83 (8.45)  Group Effect Size (Cohen’s d) at T2 d=1.98 |  | The patients randomized to CBT had much greater improvement than patients in the waitlist condition on measures of PTSD. After receiving CBT, the Delayed Treatment Group improved on this measure.  No subgroup data reported. |
|  | Paunovic 2001 | Four clients, one in the E-group and three in the CBT-group, were excluded at an early stage of treatment. The client from the E-group and two of the clients from the CBT-group missed a third consecutive appointment session. One client from the CBT-group was excluded due to hostile behaviors towards the  therapist during the first session. There were no significant differences between the four clients  who dropped out of the treatments and the 16 clients that completed the study on mean age,  duration of PTSD-symptoms, marital status, occupational status and all psychopathology measures  except the WAS. The four dropouts had significantly higher scores on the WAS (that is, more positive cognitive schemas), and were significantly more educated than the 16 clients who completed the study. | Hamilton Anxiety Scale  Exposure  T0: 44.0 (9.5)  T1: 22.9 (9.2)  T2: 22.8 (9.8)  CBT  T0: 41.0 (12.7)  T1: 21.0 (11.7)  T2: 22.1 (12.1)  ANOVA F-values  Group: 0.14  Time: 60.1*  Interaction: 0.15  Beck Anxiety Inventory  Exposure  T0: 32.8 (7.7)  T1: 14.5 (7.9)  T2: 14.9 (8.2)  CBT  T0: 37.4 (13.5)  T1: 15.1 (7.5)  T2: 16.6 (9.3)  ANOVA F-values  Group: 0.34  Time: 66.5*  Interaction: 0.54 | CAPS Severity Score  Exposure  T0: 98.4 (14.2)  T1: 46.0 (23.9)  T2: 50.5 (23.4)  CBT  T0: 95.1 (25.7)  T1: 49.0 (24.2)  T2: 52.9 (28.2)  ANOVA F-values  Group: 0.00  Time: 50.6*  Interaction: 0.20  CAPS Global Severity Score  Exposure:  T0: 3.3 (0.5)  T1: 1.8 (0.9)  T2: 1.6 (0.9)  CBT:  T0: 3.5 (0.8)  T1: 2.0 (1.1)  T2: 1.8 (1.2)  ANOVA F-values  Group: 0.29  Time: 41.4*  Interaction: 0.06  *(p<0.0001)  PTSD Symptom Scale (PSS-SR)  Exposure  T0: 34.4 (6.0)  T1: 18.1 (10.5)  T2: 18.3 (9.3)  CBT  T0: 38.9 (9.8)  T1: 16.8 (10.0)  T2: 18.5 (9.5)  ANOVA F-values  Group: 0.00  Time: 78.2  Interaction: 0.14 | Hamilton Depression Scale  Exposure  T0: 33.9 (6.3)  T1: 135. (7.3)  T2: 15.3 (8.5)  CBT  T0: 31.8 (10.6)  T1: 14.5 (8.5)  T2: 14.5 (9.5)  ANOVA F-values  Group: 0.03  Time: 57.9*  Interaction: 0.32  Beck Depression Inventory  Exposure  T0: 38.0 (5.2)  T1: 19.5 (7.3)  T2: 21.8 (8.1)  CBT:  T0: 38.0 (14.1)  T1: 15.5 (8.5)  T2: 20.0 (10.2)  ANOVA F-values  Group: 0.24  Time: 52.0*  Interaction: 0.43 | Exposure and CBT decreased the patients’ PTSD symptoms, generalized anxiety and depression after treatment. Both treatments were equally effective and there was no significant difference between them on any of the measures.  No subgroup data reported. |
| CETA | Bolton 2014 | 73 out of 347 participants were lost to follow up.  CETA:  18 discontinued intervention  1 death (not related to study)  15 could not be located  WLC:  8 discontinued participation  31 could not be located | HSCL-25 anxiety subscale  CETA vs. WLC  The mean difference in improvement from pre- to post-  Intervention was 20.48 (CI: 20.61, 20.34)  Effect size: d=0.79 | Harvard Trauma Questionnaire (HTQ)  CETA vs. WLC  The mean difference in improvement from pre- to post-  intervention was 20.43 (95% CI: 20.51, 20.35)  Effect size: d= 1.19 | HSCL-25 depression subscale.  CETA vs. WLC  Mean difference in improvement from pre- to post-intervention  was 20.49 (95% CI: 20.59, 20.40),  Effect size: d= 1.16. | Compared to a WLC comparison condition, CETA was effective in reducing symptoms of depression, PTS, and anxiety.  Subgroup analyses:  Although the study was not powered to explore differences,the results suggest that there are no statistically significant differences in effect for men and women on any of the outcome measures.  Likewise, results suggest that intervention effects for the more severely affected sample (n = 112 participants) were not qualitatively different from those of the whole sample for depression (d = 1.44), PTS (d= 1.61), or, anxiety (d= 1.05). |
| IPT | Meffert 2014 | Among the 22 randomized, 20 completed the protocol. There were  no adverse events. One participant withdrew because her husband forbade her to continue. One dropped out secondary to time  constraints. |  | Harvard Trauma Questionnaire (HTQ)  For the IPT group, mean PTSD symptoms on the HTQ decreased by approximately 40%, compared with a decrease of approximately  9% in the waitlist control group.  Effect size of IPT treatment: 2.52. | Beck Depression Inventory—II (BDI–II)  Mean depression symptoms on the BDI–II decreased by 17.1 points, or 63% in the IPT treatment group, compared with a decrease of 4.25 points,  or 16%, in the waitlist control group.  Effect size of IPT treatment: 2.38. | IPT was successful in decreasing symptoms of PTSD and depression compared to WLC.  No subgroup data reported. |
| NET | Adenauer 2011 | Loss of data sets of 15 participants. These dropouts were mostly related to participants’ refusal of MEG assessment, poor quality MEG data, and participants’ inability to complete the study as a result of deportation due to a denial of asylum by the German authorities (n=3). Importantly, these participants did not differ in any clinical variable compared to the study completers. Moreover, none of the participants dropped out of NET treatment voluntarily. |  | Clinician Administered PTSD Scale (CAPS)  NET  Pre: 88.0 (12.5)  Post: 52.8 (18.8)  p<0.001  WLC  Pre: 72.0 (13.8)  Post: 87.9 (18.5)  p=0.11 | Hamilton Depression Scale (HDRS)  NET  Pre: 25.8 (7.9)  Post: 14.9 (5.5)  p<0.001  WLC  Pre: 27.4 (5.6)  Post: 27.9 (7.4)  p=0.99 | Narrative Exposure Therapy was effective for the treatment of PTSD symptoms and comorbid depression in traumatized survivors of war and torture.  No subgroup data reported. |
|  | Hensel-Dittman 2011 | Two patients in the NET group dropped out after the first session before any exposure treatment was carried out.The third patient in the NET group dropped out after 4 sessions. The patient went into hiding from the police because of a realistic fear of deportation.  The 2 patients who dropped out from SIT showed increasingly less treatment motivation and failed to attend sessions repeatedly so that treatment could not be completed. |  | Clinician-Administered PTSD Scale (CAPS)  NET  T0: 96.47 (15.89)  T1: 76.73 (26.19) d=0.91  T2: 72.33 (18.10) d=1.42  T3: 64.12 (80.14) d=1.59  SIT:  T0: 85.15 (12.95)  T1: 82.60 (18.80) d=0.16  T2: 82.70 (26.16) d=0.12  T3: 80.14 (33.88) d=0.19 | Hamilton Depression Scale (HAM-D)  NET  T0: 29.64 (6.73)  T1: 24.70 (8.13) d=0.66  T2: 20.25 (7.52) d=1.32  T3: 21.12 (10.27) d=0.98  SIT:  T0: 26.54 (8.59)  T1: 28.10 (9.93) d=-0.17  T2: 25.60 (10.21) d=0.1  T3: 24.57 (11.415) d=0.19 | NET led to a significant PTSD symptom reduction between pretest and the 6-month follow-up as well as between pretest and the 1-year follow-up. No significant changes in PTSD symptom severity occurred in the SIT group. No significant main effect was found for the HAM-D score, for treatment, or for the time-treatment interaction. |
|  | Hijazi 2014 | 39 of the 41 participants (95.1%) assigned to brief NET  completed all three sessions. (One participant became employed after randomization and could not participate in treatment, and another completed only one session.)  Most participants (n = 53; 84.1%) completed both follow-up  assessments, but nine participants completed only the 2-month (n = 6) or 4-month (n = 3) assessment. The two conditions did  not differ on the percentage of participants missing a follow-up, and participants who completed both follow-ups did  not differ on demographics or baseline values from participants  who missed a follow-up. |  | Posttraumatic Growth Inventory (PTGI)  NET  T0:45.78 (23.85)  T1:51.22 (25.40) d=0.23  T2: 28.18 (22.44) d= 0.52**  WLC  T0: 47.68 (22.66)  T1: 41.86 (21.73) d=-0.26  T2: 39.58 (13.53) d= -0.36  Condition x Time Effect Sizes  T1: 0.48*  T2: 0.83***  HTQ Part A and Part D  NET  T0: 2.79 (0.49)  T1: 2.60 (0.66) d=0.39**  T2: 2.55 (0.66) d=-0.50**  WLC  T0: 2.76 (0.44)  T1: 2.76 (0.48) d=-0.01  T2: 2.65 (0.52) d= -0.26  Condition x Time Effect Sizes  T1: -0.48*  T2:- 0.32  *P<0.05  **p<0.01 | Beck Depression Inventory-II  NET  T0:33.91 (10.46)  T1: 27.46 (13.54) d=-0.62***  T2:25.08 (13.27) d=-0.84***  d=  WLC  T0: 33.45 (11.45)  T1: 31.45 (12.08) d= -0.17  T2: 27.38 (10.85) d=-0.53**  Condition x Time Effect Sizes  T1: -0.46*  T2: -0.27  ** p<0.01  ***p<0.001 | Compared to waitlist controls, receiving three sessions of brief  NET increased posttraumatic growth. Brief NET also reduced posttraumatic stress and depression symptoms after  2 months, with medium-sized effects. Even though symptoms  continued to decrease to the 4-month assessment, the control  condition’s symptoms improved at 4 months, eliminating the difference between conditions  No subgroup data reported. |
|  | Neuner 2004 | Only in the supportive counseling group did any patients fail to complete the full treatment; 2 patients in this group discontinued treatment. The reason for not participating or for terminating treatment was generally due to a lack of time given the need to cultivate the fields at the beginning of the  rainy season. | Self-Reporting Questionnaire 20*  NET  T0: 0.27 (0.12)  T1: 0.36 (0.19)  T2: 0.38 (0.12)  T3: 0.44 (0.19)  Effect size T0 to T1 = -0.6  Effect size T0 to T3 = -1.1  SC  T0: 0.34 (0.11)  T1: 0.33 (0.21)  T2: 0.33 (0.14)  T3: 0.36 (0.14)  Effect size T0 to T1 = 0.1  Effect size T0 to T3 = 0.1  PE  T0: 0.23 (0.15)  T1: 0.33 (0.19)  T2: 0.37 (0.14)  T3: 0.35 (0.17)  Effect size T0 to T1 = -0.7  Effect size T0 to T3 = -0.8  *used to indicate comorbid symptoms of anxiety and depression.  SC = Supportive Counseling  PE = Psychoeducation | Posttraumatic Stress Diagnostic Scale  NET  T0: 25.2 (7.4)  T1: 19.1 (11.7)  T2: 24.5 (7.8)  T3: 16.0 (5.1)  Effect size T0 to T1 = 0.6  Effect size T0 to T3 = 1.6  SC  T0: 22.0 (8.0)  T1:19.8 (10.9)  T2:22.8 (10.1)  T3:23.1 (7.7)  Effect size T0 to T1 = 0.2  Effect size T0 to T3=-0.1  PE  T0:19.5 (8.0)  T1: 21.2 (9.4)  T2: 27.7 (6.6)  T3: 23.9 (7.0)  Effect size T0 to T1 = -0.5  Effect size T0 to T3 = -0.9  Composite International Diagnostic Interview—PTSD part  NET  T0: 13.4 (2.1)  T1: --  T2: --  T3: 8.9 (2.7)  Effect size T0 to T1 --  Effect size T0 to T3 = 1.9  SC  T0:13.9 (2.3)  T1:--  T2: --  T3: 12.6 (3.2)  Effect size T0 to T1 --  Effect size T0 to T3 = 0.4  PE  T0: 14.2 (2.9)  T1: --  T2: --  T3: 13.4 (3.3)  Effect size T0 to T1 --  Effect size T0 to T3 = 0.3  Diagnosis of PTSD at T3  NET n=4/14 (29%)  SC n=11/14 (79%)  PE n=8/10 (80%)  SC = Supportive Counseling  PE = Psychoeducation | *See *Anxiety* | At 1-year follow-up, the narrative exposure therapy group presented with a significantly better outcome on measures of post-traumatic stress than either of the other groups. All groups improved somewhat during the 1-year period after therapy, according to indicators of anxiety and depression,but effect sizes did not indicate a superiority of the narrative exposure therapy group on these measures. |
|  | Neuner 2008 | In the NET group, 4 participants (3.6%) refused or dropped out of treatment, whereas in the TC group 22 participants (19.8%) did not complete therapy. The difference  was significant. Dropouts/refusers did not differ significantly from treatment completers in age, nationality, possessions, number of event types, or pretest PDS and health scores |  | The Posttraumatic Stress Diagnostic Scale (PDS)  NET  T0: 25.9 (12.2)  T1: 5.4 (6.6)  T2: 6.1 (6.8)  Effect size T0 to T1 = 1.4  Effect size T0 to T2 = 1.4  TC  T0:26.7 (12.5)  T1: 5.3 (5.7)  T2: 5.0 (6.6)  Effect size T0 to T1 = 1.5  Effect size T0 to T2 = 1.5  MG  T0: 21.3 (10.6)  T1: =--  T2:10.1 (8.1)  Effect size T0 to T1 --  Effect size T0 to T2 = 0.8  TC = Trauma counseling  MG = Monitoring group |  | Over the period of 6 –9 months,  the groups that received active treatment developed statistically  and clinically better on scales of PTSD than did a non treated  monitoring group.The comparison of NET and TC showed no significant difference between the approaches in reducing PTSD symptoms.However, significantly fewer participants dropped out of the NET treatment. |
|  | Neuner 2010 | Two patients from the NET group but no  patient from the TAU group dropped out of the study. One NET treatment could not be carried out according to the manual because a severe  hyperventilation syndrome interfered with narrative exposure. This patient was excluded  from the study. Another patient dropped out of  the NET group before the first exposure session because he refused to continue. Both patients were not available for follow-up examination. |  | Posttraumatic Stress Diagnostic Scale (PDS)  NET  T0: 38.9 (6.4)  T1: 26.0 (9.2)  Effect size: 1.6  TAU  T0: 36.9 (8.0)  T1: 34.1 (6.1)  Effect size 0.4 | The Hopkins Symptom Checklist – 25 (HSCL-25) Depression scale  NET  T0: 3.0 (0.4)  T1: 2.6 (0.6)  Effect size: 0.8  TAU  T0: 3.0 (0.5)  T1: 2.9 (0.5)  Effect size: 0.2 | Significantly better trajectory for  NET patients than TAU patients in the 6 months after treatment in terms of PTSD symptoms but no difference related to depression symptoms. |
|  | Sternmark et al. 2013; Halvorsen et al. 2014 | In the NET group, 33/51 participants completed the study. Participants were lost to follow up because they moved within Norway (n=4), were sent out of the country (n=3), quit due to treatment stress (n=1) or dropped out.  In the TAU group, 21/30 participants completed the study. Participants were lost to follow up because they moved within Norway (n=2), were sent out of the country (n=2), or dropped out. |  | Clinician-Administered PTSD Scale (CAPS)  NET  Refugees within-group effect size  T1: 1.37 (95%CI 0.70 - 2.04)  T2: 1.53 (95%CI 0.85 - 2.22)  Asylum seekers within-group effect size  T1: 0.90 (0.06-1.74)  T2: 0.93 (0.09-1.77)  TAU  Refugees within-group effect size  T1: 0.42 (95%CI -0.33-1.16)  T2: 0.57 (95%CI -0.19-1.33)  Asylum seekers within-group effect size  T1: 0.82 (95%CI -0.28-1.91)  T2: 0.31 (95%CI -0.75-1.36) | Hamilton Rating scale for Depression (HAM-D)  NET  Refugees within-group effect size  T1: 0.89 (95%CI 0.26 - 1.52)  T2: 1.07 (95%CI 0.42-1.71)  Asylum seekers within-group effect size  T1: 0.72 (95%CI -0.11 - 1.54)  T2: 0.31 (95%CI -0.49 - 1.12)  TAU  Refugees within-group effect size  T1: 0.60 (95%CI -0.16-1.36)  T2: 0.57 (95%CI -0.18 - 1.33)  Asylum seekers within-group effect size  T1: 1.10 (95%CI -0.03 - 2.22)  T2: -0.32 (95%CI -1.37 - 0.74) | Both refugees and asylum  seekers reduced their mental health problems related to PTSD and depression by the treatment provided by the general psychiatric services. The symptom reductions in PTSD and depression were more pronounced in the patients who received NET. Refugees with  a secure legal status reported less depression across all time-points.  However, both asylum seekers and refugees profited from the  treatment to the same degree in spite of their differences at the  outset with regard to the symptom levels. Likewise, the groups of refugees and asylum seekers who were given NET, emerged with a bigger fall in diagnostic status for PTSD than those who were given TAU. |
| SM | Carlsson 2018 | Of 140 allocated, 14 participants were withdrawn from the study due to: psychiatric/somatic hospitalization (n=2); wrongly included (n=5), stopped treatment and withdrew consent (n=6), received wrong treatment (n=1).  21 participants received less than 8 sessions of the allocated psychotherapy. | Hopkins Symptom Checklist-25 (HSCL-25)*  SM  T0: 3.05 (0.05)  T1: 2.96 (0.08)  Effect size: 0.20 p=0.19  CR  T0: 3.06 (0.06)  T1: 3.01 (0.08)  Effect size: 0.11 p=0.45  Hamilton Anxiety Ratings Scale (HAM-A)  SM  T0: 24.12 (0.95)  T1: 24.78 (1.25)  Effect size: 0.32 p=0.03  CR  T0: 26.09 (0.95)  T1: 27.17(1.24)  Effect size: 0.15 p=0.35  *Assesses symptoms of anxiety and depression  CR = Cognitive Restructuring | Harvard Trauma Questionnaire (HTQ), part IV.  SM  T0: 3.18 (0.06)  T1: 3.10 (0.08)  Effect size: 0.19 p=0.19  CR  T0: 3.21 (0.05)  T1: 3.06 (0.07)  Effect size: 0.35 p=0.03  CR = Cognitive Restructuring | Hamilton Depression Rating Scale (HAM-D)  SM  T0: 23.83 (0.83)  T1: 21.98 (1.10)  Effect size: 0.29 p=0.04  CR  T0: 24.34 (0.78)  T1: 23.60 (0.97)  Effect size: 0.43 p=0.12  CR = Cognitive Restructuring | No difference between SM and CR on PTSD symptoms, but a possible advantage of SM on anxiety. |
