# Supplementary material for: Understanding Supporting and Hindering Factors in Community-Based Psychotherapy for Refugees: A Realist-Informed Systematic Review
Source: Int J Environ Res Public Health. 2020 Jun 27;17(13):4618. doi: 10.3390/ijerph17134618 (PMC7369747; doi:10.3390/ijerph17134618)
Supplement: Supplementary file 1 [file ijerph-17-04618-s001.zip › S4File_Critical Appraisal.docx]

**Supplementary File S4: Critical Appraisal**

**Summary:** In our risk of bias assessment, all included studies reported randomized allocations. Only three studies reported allocation concealment. The majority of studies had methodological deficiencies in the blinding of participants, personnel and outcome assessors. Three studies failed to blind participants and personnel. Outcome assessors were not blinded in five studies. One study reported that missing data affected outcome estimates. Study protocols were obtained in eight studies and the rest reported all outcome measures. One study was judged as low relevancy, 5 were judged as medium relevancy, and the remaining 8 studies were judged as high relevancy.

**Cochrane Risk of Bias**


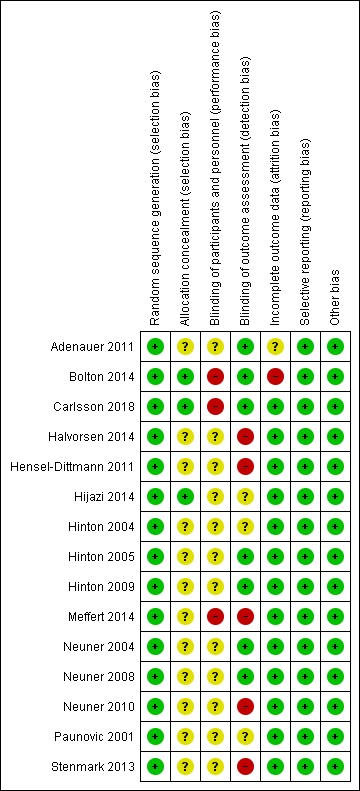


**Assessments of relevancy**

| **Study** | **Does the study provide information on context? (Y/N)** | **Does the study provide information on mechanisms? (Y/N)** | **Rating** |
| --- | --- | --- | --- |
| Adenauer et al. 2011 | Yes | Yes | High |
| Bolton et al. 2014 | Yes | Yes | High |
| Carlsson et al. 2018 | Yes | No | Medium |
| Hensel-Dittman et al. 2011 | Yes | Yes | High |
| Hijazi et al. 2014 | Yes | Yes | High |
| Hinton et al. 2009 | Yes | Yes | High |
| Hinton et al. 2005 | Yes | Yes | High |
| Hinton et al. 2004 | No | No | Low |
| Meffert et al. 2014 | Yes | No | Medium |
| Neuner et al. 2010 | Yes | Yes | High |
| Neuner et al. 2008 | Yes | No | Medium |
| Neuner et al. 2004 | Yes | Yes | High |
| Paunovic et al. 2001 | No | Yes | Medium |
| Sternmark et al. 2013; Halvorsen et al. 2014 | No | Yes | Medium |
