# Supplementary material for: Understanding Supporting and Hindering Factors in Community-Based Psychotherapy for Refugees: A Realist-Informed Systematic Review
Source: Int J Environ Res Public Health. 2020 Jun 27;17(13):4618. doi: 10.3390/ijerph17134618 (PMC7369747; doi:10.3390/ijerph17134618)
Supplement: Supplementary file 1 [file ijerph-17-04618-s001.zip › S3Table_Search Strategy.docx]

**Table S3:  Search Strategy**

| 1 (refugee* or asylum seeker*).ti,ab,kw.  2 Refugees/  3     Undocumented Immigrants/  4 1 or 2 or 3  5 Psychological Trauma/  6 Post?traumatic stress disorder.ti,ab,kw.  7 ptsd.ab,ti,kw.  8     trauma*.ti,ab,kw,tw.  9 Stress Disorders, Post-Traumatic/  10 or/5-9  11 4 and 10  12 (diagnos?s or therap* or treatment*).mp.  13 Psychotherapy/  14 12 or 13  15 11 and 14  16 clinical study.pt.  17 controlled clinical trial.pt.  18 randomized controlled trial.pt.  19 pragmatic clinical trial.pt.  20 random*.ab.  21 review.pt,ti.  22     meta-analysis.pt,ti.  23 or/16-22  24 15 and 23  25 limit 24 to yr="2000 -Current"  26 remove duplicates from 25 |
| --- |
