# Supplementary material for: Understanding Supporting and Hindering Factors in Community-Based Psychotherapy for Refugees: A Realist-Informed Systematic Review
Source: Int J Environ Res Public Health. 2020 Jun 27;17(13):4618. doi: 10.3390/ijerph17134618 (PMC7369747; doi:10.3390/ijerph17134618)
Supplement: Supplementary file 1 [file ijerph-17-04618-s001.zip › S2Table_Inclusion Exclusion Criteria.docx]

**Table S2: Inclusion and Exclusion Criteria**

| **Study Characteristics** | **Inclusion Criteria** |  |
| --- | --- | --- |
| Population | Adult male or female refugees and/or asylum seekers with experience of trauma. Diagnosis of PTSD or other mental health conditions not required. | |
| Interventions | Any talk-based psychotherapy (e.g. CBT, NET) delivered by any type of provider in any setting. | |
| Comparison | No intervention, standard intervention, alternative intervention, treatment as usual, waitlist control. | |
| Outcomes | Symptoms of PTSD, depression or anxiety. | |
| Study Characteristics | Randomized control trial  Conducted in any geographic context  Published between 2000 and 2018  No language restrictions | |
| **Study Characteristics** | **Exclusion Criteria** | **Justifications** |
|  | Children and adolescents    Group therapy | Treatment approaches for children can differ substantially from treatment for adults. Special considerations and training in child development are necessary and are not generalizable to adult populations.  The focus of this review is on primary care. Primary care visits most frequently occur on an individual basis. |
